# Supplementary material for: Bioinformatic Surveillance Leads to Discovery of Two Novel Putative Bunyaviruses Associated with Black Soldier Fly
Source: Viruses. 2023 Jul 29;15(8):1654. doi: 10.3390/v15081654 (PMC10460066; doi:10.3390/v15081654)
Supplement: Supplementary file 1 [file viruses-15-01654-s001.zip › viruses-2499178-supplementary.pdf]

viruses

MDPI

Supplementary Materials:

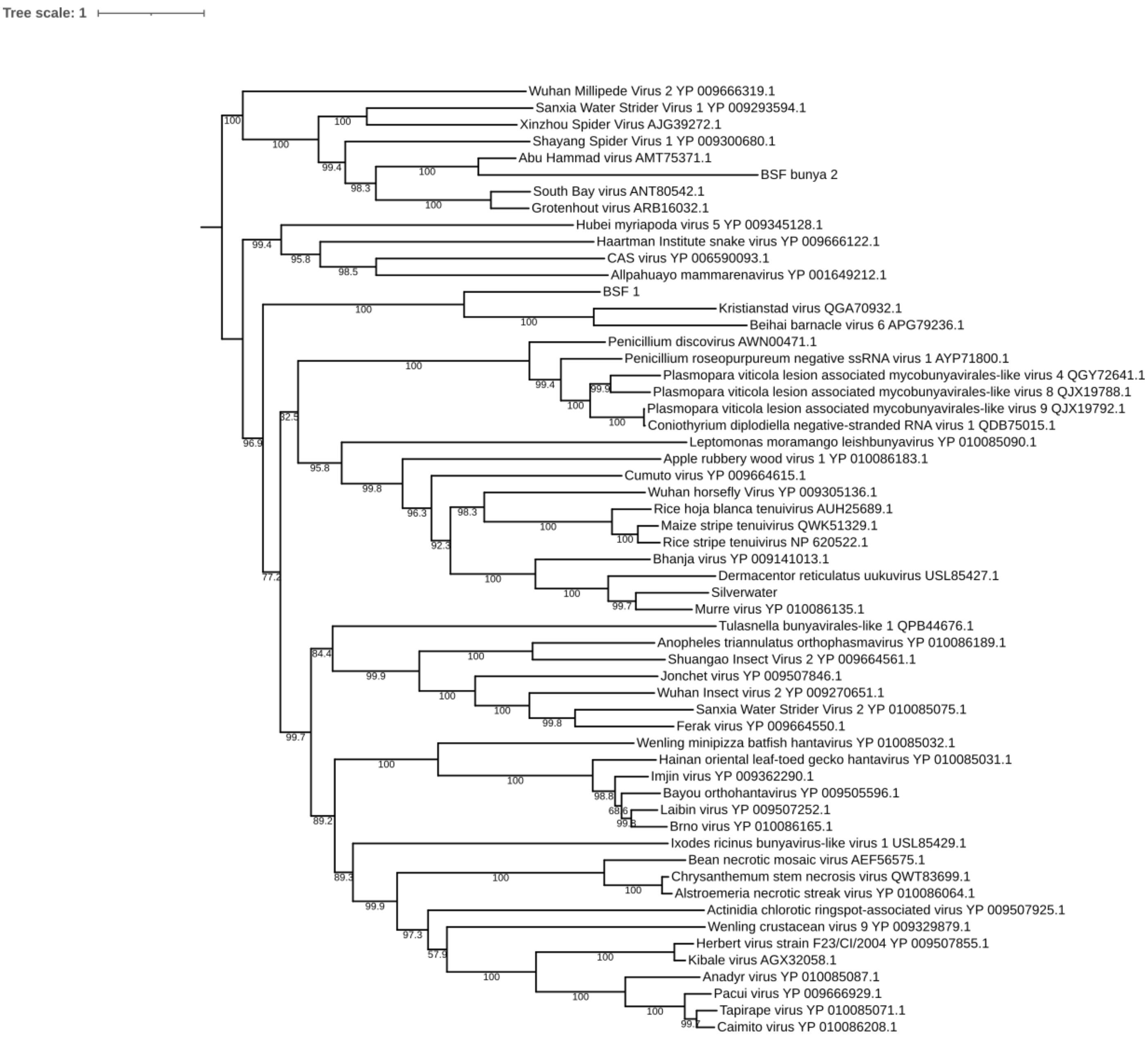

Figure S1. Fully uncollapsed bunyavirus phylogenetic tree of the Bunyavirus RdRps used in this study. This figure is the same tree shown in Figure 1, but all nodes are uncollapsed. All bootstrap values are shown.

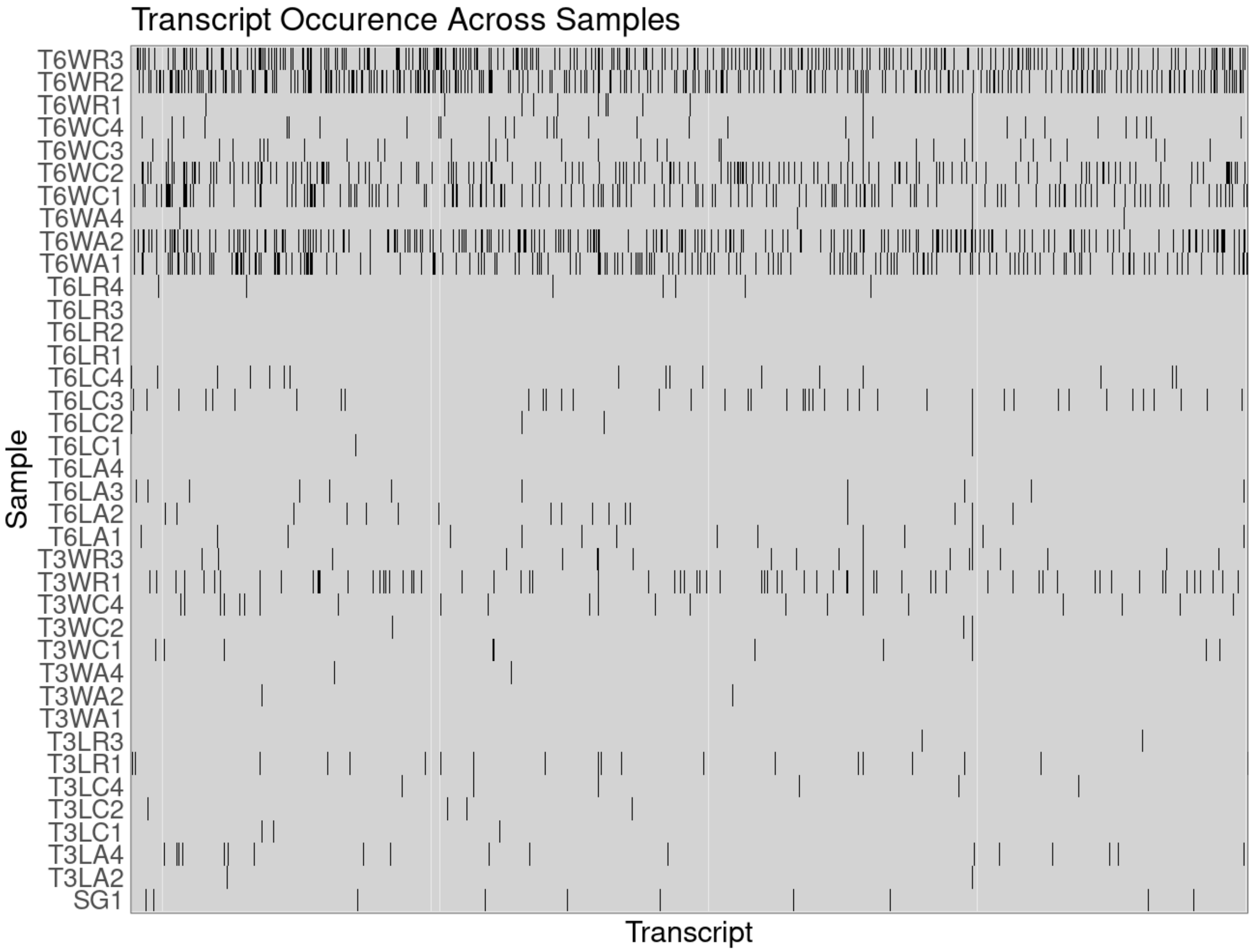

Figure S2. Transcript co-occurrence across samples. Occurrence matrix of all CD-hit transcript clusters across samples, where black indicates presence and grey indicates absence. The X axis is all transcript clusters from CD-hit at 95% sequence identity.

Table S1. Top five BLAST hits of the two novel bunyavirus L segments discovered in this study. The Table contains Each BLAST hit, the subject's accession number, the corresponding e-value, and the host from which the virus was identified. Notably, all hosts are arthropods.

**BSF Uncharacterized bunyavirus-like 1 (5696 nt)**				**BSF Nairovirus-like 1 (4543 nt)**			
BLAST Hit	Accession	E-value	Host	BLAST Hit	Accession	E-value	Host
RdRp [Beihai barnacle virus 6]	APG79236.1	2E-115	Barnacle	RdRp [Beihai barnacle virus 6]	APG79236.1	1E-63	Barnacle
RdRp [Bunyavirales sp.]	QTW97784.1	1E-113	Mosquito	RdRp [Kristianstad virus]	QGA70932.1	2E-54	Mosquito
RdRp [XiangYun bunya-arena-like virus 10]	UUG74075.1	1E-107	Mosquito	RdRp [Jiangxia Mosquito Virus 1]	AJG39240.1	3E-54	Mosquito
RdRp [Jiangxia Mosquito Virus 1]	AJG39240.1	2E-106	Mosquito	RdRp [Bunyavirales sp.]	QTW97784.1	1E-52	Mosquito
RdRp [Kristianstad virus]	QGA70932.1	3E-106	Mosquito	RdRp [XiangYun bunya-arena-like virus 10]	UUG74075.1	1E-46	Mosquito

Table S2. Putative microbe-associated viruses found within BSF and frass samples. The table only contains contigs with a length greater than 2000.

Transcript	Length (nt)	Closest BLAST Hit	evalue	Putative Type
T6WA2_S51_DN382_c0_g2_i1	3554	YP_010769592.1: ssRNA phage SRR6960799_20	0.0	Levivirus (+ssRNA)
T6WR2_S83_DN3594_c0_g1_i1	3472	URG16190.1: Leviviridae sp.	0.0	Levivirus (+ssRNA)

Table S3. SRA data that were positive for BSF uncharacterized bunyavirus-like 1. The table contains the SRA accession number along with the type of sample the virus RdRp transcript was found in.

SRA Accession	Sample Type
SRR9068902	BSF Frass
SRR9068904	BSF Frass

SRR9068905	BSF Frass
SRR9068906	BSF Frass
SRR9068923	Starved BSF Larvae
SRR9068924	Starved BSF Larvae

Table S4. **List of all SRA data used in this study by accession number.**

ERR1801985
ERR1801986
ERR1801987
ERR1801988
ERR1801989
ERR1801990
ERR1801991
ERR1801992
ERR1801993
ERR1801994
ERR1801995
ERR1801996
ERR1801997
ERR1801998
SRR10158821
SRR10233312
SRR14339782
SRR14339783
SRR14339784
SRR14339785
SRR14339786
SRR14339787
SRR14339788
SRR14339789
SRR14339790
SRR14339791
SRR14339792
SRR14339793
SRR14339794
SRR14339795
SRR14339796
SRR18283674
SRR18283675
SRR18283676

SRR18283677
SRR18283678
SRR18283679
SRR18283680
SRR18283681
SRR18283682
SRR18283683
SRR18283684
SRR18283685
SRR18283686
SRR18283687
SRR18283688
SRR18283689
SRR18283690
SRR18283691
SRR18283692
SRR18283693
SRR18283694
SRR18283695
SRR18283696
SRR18283697
SRR18283698
SRR18283699
SRR18283700
SRR18283701
SRR18283702
SRR18283703
SRR18283704
SRR18283705
SRR18283706
SRR6656085
SRR6656086
SRR6656087
SRR6656088
SRR8242276
SRR8242277
SRR8242278
SRR8242279
SRR8242280
SRR8242281
SRR8242282
SRR8242283

SRR8242284
SRR8242285
SRR8242286
SRR8242287
SRR8242288
SRR8242289
SRR8242290
SRR8242291
SRR8242292
SRR8242293
SRR8242294
SRR8242295
SRR8242296
SRR8242297
SRR8242298
SRR8242299
SRR9068902
SRR9068904
SRR9068905
SRR9068906
SRR9068907
SRR9068908
SRR9068909
SRR9068910
SRR9068911
SRR9068912
SRR9068913
SRR9068914
SRR9068915
SRR9068916
SRR9068917
SRR9068918
SRR9068919
SRR9068920
SRR9068921
SRR9068922
SRR9068923
SRR9068924
SRR9068925
SRR9068926
